# Prognostic value of neutrophil-to-lymphocyte ratio in breast cancer

**DOI:** 10.1016/j.fob.2015.05.003

**Published:** 2015-05-12

**Authors:** Jie Chen, Qiwen Deng, Yuqin Pan, Bangshun He, Houqun Ying, Huiling Sun, Xian Liu, Shukui Wang

**Affiliations:** a Department of Life Sciences, Nanjing Normal University, Nanjing, Jiangsu, China; b Central Laboratory, Nanjing First Hospital, Nanjing Medical University, Nanjing, Jiangsu, China; c Medical College, Southeast University, Nanjing, Jiangsu, China

**Keywords:** NLR, neutrophil-to-lymphocyte ratio, OS, overall survival, DFS, disease-free survival, RFS, recurrence-free survival, CSS, cancer specific survival, HR, hazard ratio, CI, confidence interval, Breast cancer, Inflammation, NLR, Prognosis

## Abstract

Inflammation is an essential component of pathogenesis and progression of cancer. A high neutrophil-to-lymphocyte ratio (NLR) is considered as a prognostic indicator for breast cancer. This meta-analysis was conducted to establish the overall accuracy of the NLR test in the diagnosis of breast cancer. A comprehensive search of the literature was conducted by using PubMed, Web of Science and China National Knowledge Infrastructure (CNKI). Published studies dating up to July 2014 and 4,293 patients were enrolled in the present study. In order to evaluate the association between NLR and overall survival (OS), disease-free survival (DFS), recurrence-free survival (RFS) or cancer specific survival (CSS), the hazard ratios (HRs) and their 95% confidence intervals (CIs) were extracted. OS was the primary outcome. The results suggested that increased NLR was a strong predictor for OS with HR of 2.28 (95% CI = 1.08–4.80, *P_heterogeneity_ *< 0.001). Stratified analyses indicated that a high NLR appeared to be a negative prognostic marker in Caucasian populations (HR = 4.53, 95% CI = 3.11–6.60, *P_heterogeneity_* = 0.096), multivariate analysis method (HR = 2.10, 95% CI = 1.52–2.89, *P_heterogeneity_ *= 0.591), and mixed metastasis (HR = 4.53, 95% CI = 3.11–6.60, *P_heterogeneity_ *= 0.096). Elevated NLR was associated with a high risk for DFS (HR = 1.38, 95% CI = 1.09–1.74, *P_heterogeneity_ *= 0.050) and in subgroups of multivariate analysis (HR = 1.64, 95% CI = 1.25–2.14, *P_heterogeneity_ *= 0.545) and mixed metastasis (HR = 1.99, 95% CI = 1.28–3.09, *P_heterogeneity_ *= 0.992). In summary, NLR could be considered as a predictive factor for patients with breast cancer.

## Highlights

• A literature search was conducted using PubMed, Web of Science and CNKI.
• Increased NLR was a strong predictor for overall survival and disease-free survival.
• Subgroup analyses stratified by ethnicity, analysis method and metastasis were conducted.
• NLR could be considered as a predictive factor for patients with breast cancer.

## 1 Introduction

Breast cancer is a common malignancy that affects the health of women worldwide. One in eight women will be diagnosed with breast cancer in their lifetime [1]. 5–7% of women are diagnosed before the age of 40, and the highest frequency is found in the age group 25 to 39 [2–5]. With the rapid advancement of early diagnosis and treatment in breast cancer, more than four fifths of patients are now successfully treated [4] and the mortality has recently declined in young women [6]. However, a large proportion of patients were still suffered from breast cancer due to heterogeneity of diagnosis and treatment. Therefore, it is crucial to understand causes contributing to breast carcinogenesis, invasion and metastasis and to identify effective early-diagnostic and prognostic biomarkers that help to diagnose, evaluate treatment efficacy and prognosis and follow-up schedule [7].

It has been demonstrated that the inflammatory response plays an important role in the development and progression of various cancers, including breast cancer [8–10]. The cancer-related inflammatory response helps proliferation and survival of malignant cells, angiogenesis and metastasis of breast cancer, and it subverts adaptive immune responses and alters responses to chemotherapeutic agents. Severe inflammatory responses result in a weaker adaptive immune response, leading to an imbalance of immune response and malignant cancer to promote cancer progression and poor OS.

Biomarkers such as neutrophils, lymphocytes, neutrophil-to-lymphocyte ratio (NLR), mean platelet volume, red cell distribution width, circulating tumor cells and gamma-glutamyl transferase have been proposed as potential prognostic factors for cancer [11–15]. There is accumulating evidence for the association of NLR with survival of patients with many kinds of cancers, including breast cancer [16–23]. However, the published results are inconsistent. Some studies reported that NLR was significantly associated with shorter DFS and OS in breast cancer patients [24,25], while others showed that NLR could not be considered as an independent prognostic factor for breast cancer [26,27].

In order to obtain an objective and consistent conclusion, we therefore conducted this comprehensive systematic review and meta-analysis of the association between NLR and survival of breast cancer.

## 2 Materials and methods

### 2.1 Search strategy

This meta-analysis was conducted according to Preferred Reporting Items for Systematic Reviews and Meta-Analyses (PRISMA) statement and methods [28,29]. A comprehensive literature search was carried out using search terms of “neutrophil-to-lymphocyte ratio (NLR)”, “breast cancer or tumor or carcinoma” and “prognosis or outcome or survival” in databases of PubMed, Web of Science and CNKI dating up to July 2014. Hand searches were performed to obtain substantial relevant study by reviewing all references within all relevant articles. All selected literatures were journal articles in Chinese and English. This study was approved by the institution ethics committee of Nanjing Normal University.

### 2.2 Selection criteria

In the meta-analysis, studies were considered eligible if they met the following criteria: (1) study investigated the association between NLR and clinical prognosis in patients with breast cancer; (2) study provided sufficient data for estimating hazard ratio (HR) with 95% confidence interval (CI). Meanwhile, studies were excluded based on the following criteria: (1) duplicate publications; (2) insufficient data for further analysis; (3) letters, reviews, meeting abstracts, editorials, and case reports; (4) other topics.

### 2.3 Data extraction

The following data, the first author, year of publication, name of journal, county of origin, ethnicity of the study population, type of specimen, metastasis, cut-off value, follow-up period, number of patients included in analysis, and HR with its 95% CI for overall survival (OS), disease-free survival (DFS), recurrence-free survival (RFS) or cancer specific survival (CSS) were extracted from each eligible study by two independent investigators (JC, QWD). If there was any disagreement, it solved by discussion to reach a consensus.

### 2.4 Statistical analysis

HR and its 95 % CI were selected as common measurements to assess the strength of the association between NLR and prognosis in breast cancer. Cochran’s Q test was chosen to evaluate the heterogeneity and Higgins I-squared statistic was carried out to estimate the degree of heterogeneity of pooled results. The random-effect and fixed-effect models were used to calculate the pooled HR and its 95% CI. If *P*_H_ < 0.05, the random-effect model (DerSimonian–Laird method) was applied to calculate the pooled HRs [30]. Otherwise, the fixed-effect model (Mantel–Haenszel method) was employed [31]. The HR is commonly and conveniently estimated via a Cox proportional hazards model, which can include potential confounders as covariates. HR > 1 reflects that elevated NLR is associated with the corresponding variate, while HR < 1 has the opposite meaning. Furthermore, subgroup was performed to explore the heterogeneity among studies which stratified by ethnicity, analysis method and metastasis. Sensitivity analysis was conducted to check whether individual study influenced the results by sequential omission of each study in this meta-analysis. Additionally, Begg’s funnel plot and Egger’s linear regression test were used to assess the extent of publication bias in the meta-analysis and *P*_E_ < 0.05 was considered as statistically significant. Statistical analysis was performed by Stata 11.0 software (STATA Corporation, College Station, TX, USA).

## 3 Results

### 3.1 Included studies

**Fig. 1:**
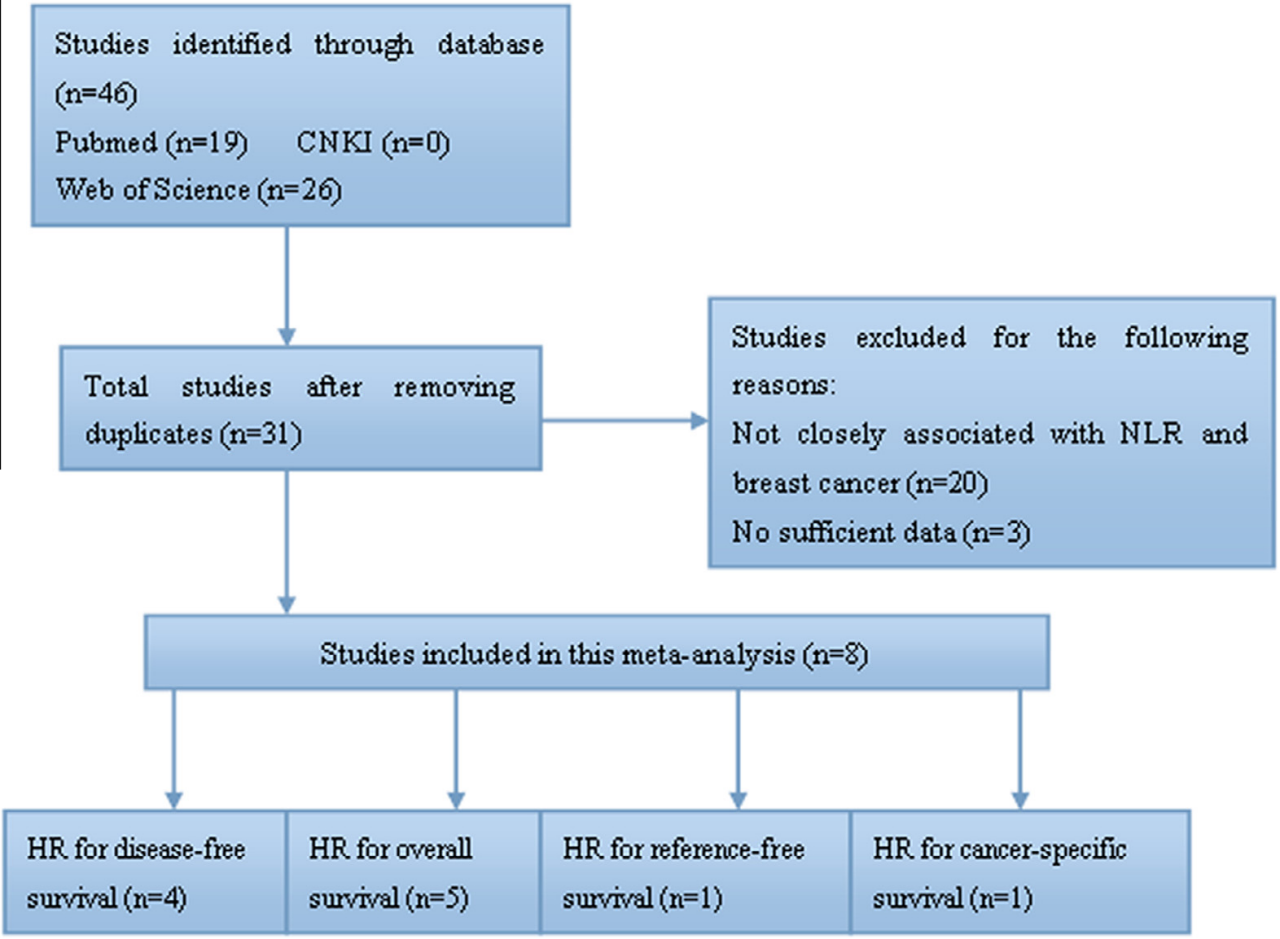
Flow diagram of studies included in this meta-analysis.

A total of 45 potentially relevant articles were retrieved. 14 papers were defined duplicate publications according to their titles. Then 20 articles were excluded because of obvious lack of relevance. A careful review of the remaining 11 studies revealed that 3 studies did not provide sufficient information. Finally, 8 studies were included in the meta-analysis (Fig. 1) [16,22–25,27,32,33].

### 3.2 Study characteristics

The main features of eligible studies were shown in Table 1. The eligible studies were published in a period of 2012 to 2014 and contained a total of 4,293 patients. In total, 8 studies were enrolled and 4 studies were conducted in Asian and Caucasian population, respectively. 5 studies were involved in mixed metastasis and the others without metastasis. The cut-off values applied in the studies were not consistent and it was not provided in one study [16]. Among them, 5, 4, 1 and 1 studies investigated the relationship of NLR and OS, DFS, RFS, and CSS, respectively. The useful data of HRs and 95% CIs were obtained from multivariate analysis in 5 studies and univariate analysis in 3 studies, respectively.

### 3.3 Overall survival

**Fig. 2:**
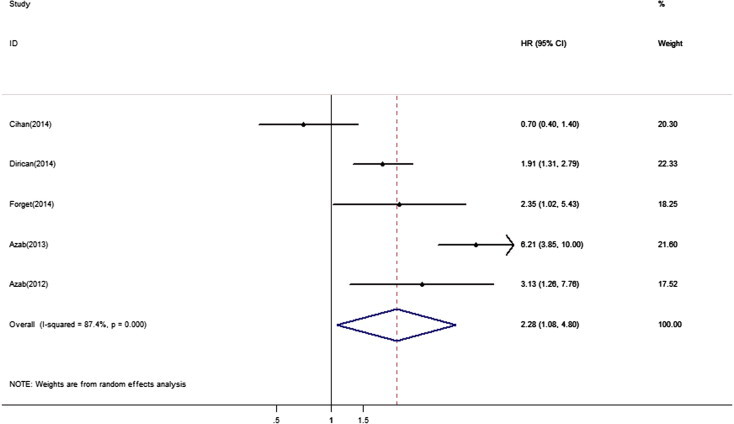
Forest plots of studies evaluating hazard ratios (HRs) of NLR for overall survival. The solid diamond represents each individual study and the hollow diamond represents overall studies. Error bars are 95% confidence intervals.

The pooled analysis was conducted in 5 studies including 3,350 patients that reported HR for OS. The main results of this meta-analysis were listed in Table 2 and Fig. 2. The results showed that elevated NLR was associated with a worse outcome for OS with the pooled HR of 2.28 (95% CI = 1.08–4.80, *P*_H_ < 0.001). Subgroup analyses showed that the prognostic effect of NLR was found only in Caucasian population (HR = 4.53, 95% CI = 3.11–6.60, *P*_H_ = 0.096) and it was examined to be was a strong prognostic factor in multivariate analysis (HR = 2.10, 95% CI = 1.52–2.89, *P*_H_ = 0.591). When metastasis was taken into consideration, increased NLR was associated with a poor prognosis for OS in mixed metastasis (HR = 4.53, 95% CI = 3.11–6.60, *P*_H_ = 0.096).

**Fig. 3:**
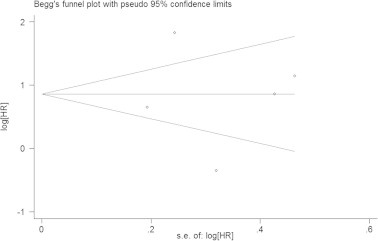
Begg’s funnel plot assessed publication bias test of the included studies for overall survival (OS). Each circle represents as an independent study for the indicated association. Log[HR], natural logarithm of HR. Horizontal lines mean effect size. HR: hazard ratio.

From sensitivity analysis we found that the result was not obviously impacted by an included study conducted by Cihan et al. [27]. The HR for it was 3.08 (95% CI = 1.59–5.96, *P*_H_ = 0.002). The shape of funnel plots showed no evidence of publication bias in the analysis (Fig. 3) and the result was further supported by Egger’s tests (*P*_E_ = 0.896).

### 3.4 Disease-free survival

**Fig. 4:**
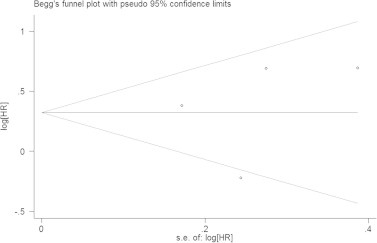
Begg’s funnel plot assessed publication bias test of the included studies for disease-free survival (DFS). Each circle represents as an independent study for the indicated association. Log[HR], natural logarithm of HR. Horizontal lines mean effect size. HR: hazard ratio.

4 studies comprising 2,764 patients were included to assess the association between NLR and DFS in breast cancer (Table 2). Overall, elevated NLR was associated with a high risk for DFS (HR = 1.38, 95% CI = 1.09–1.74, *P*_H_ = 0.050) and in subgroups of multivariate analysis (HR = 1.64, 95% CI = 1.25–2.14, *P*_H_ = 0.545) and mixed metastasis (HR = 1.99, 95% CI = 1.28–3.09, *P*_H_ = 0.992). Interestingly, the same study [27] had no effect on sensitivity analysis by removing one study each time. The HR for it was 1.64 (95% CI = 1.25–2.14, *P*_H_ < 0.001). The rest studies [24,25,27,33] might be source of heterogeneity. The Begg’s funnel plot (Fig. 4) and the Egger’s test (*P *= 0.762) did not provide any obvious evidence of publication bias.

## 4 Discussion

Inflammation has been shown to be an important factor in the development of tumorigenesis [34]. Peripheral blood tests before treatment or at the time of diagnosis could reflect inflammatory conditions within the tumor. Inflammation-related markers such as absolute white blood cell count, C-reactive protein (CRP), cytokines, platelet-to-lymphocyte ratio (PLR), and NLR have been shown to be associated with specific outcomes in cancer patients [35]. NLR is a biomarker for inflammation and it can be more easily and conveniently measured than conventional markers and at a low cost. A meta-analysis recently reported by Templeton et al. [35] only included 3 original studies and did not show a significant correlation between NLR and survival of breast cancer. The current meta-analysis combined the outcomes of 4,293 cancer patients from 8 studies was to assess the prognostic effect of NLR in breast cancer. In this meta-analysis, we found that high level of NLR significantly affected OS and DFS in breast cancer in overall population. When groups were stratified by ethnicity, analysis method and metastasis, elevated NLR predicted poor OS in Caucasian population, multivariate analysis, and mixed metastasis, respectively. Meanwhile, the significant association was observed in multivariate analysis, and mixed metastasis subgroups in DFS. These findings indicated that NLR was associated with ethnicity, analysis methods and metastasis and it could act as a prognostic biomarker in predicting clinical outcome for breast cancer.

The mechanism between the high level of NLR and poor outcome of breast cancer remained unclear. There were several possible explanations for the association between elevated NLR and poor prognosis in breast cancer. First of all, their relationship might be explained by means of an inflammation response caused by cancer cells. As is known, lymphocytes can reduce malignant progression as tumor infiltration via a series of subtypes of lymphocytes, CD3^+^ T cells, CD8^+^ T cells, Th1 CD4^+^ T cell, and p46^+^ natural killer cells, which has been shown to improve the survival of patients with malignancy [36–39]. An important event of immune escape was T-lymphocyte dysfunction. T-lymphocytes were a common kind of tumor infiltrating lymphocytes (TILs). A study suggested that anergic CD8^+^ T-lymphocytes were functionally unresponsive, unable to directly lyse melanoma target cells or produce cytokines in response to mitogen [40–42]. So, immunogenic tumor variants would take place when tumor cells were able to escape from immune surveillance. Another explanation was that IL-17 could recruit neutrophil via CXC chemokines, such as CCL2 released from IL-17-producing T cell. Therefore, IL-17-producing T cells released CXC chemokines that recruited neutrophils, leading to elevated NLR.

To the best of our knowledge, it is the largest sample size of meta-analysis to investigate the prognostic role of NLR in breast cancer. Several strong points and limitations should be addressed as follow: this is the first study to investigate the association between NLR and survival of breast cancer; furthermore, heterogeneity test was conducted to confirm the outcomes of subgroup analyses and explore sources of heterogeneity; finally, there was almost no publication bias in this meta-analysis, showing the results were reliable. However, first of all, only summarized data rather than individual patient data were pooled in our study, which might preclude us from conducting a more in-depth analysis; secondly, even though we used prospectively listed patients and high-quality databases, uncontrolled and unrecognized biases might exist; thirdly, geographical differences in the frequency of breast cancer subtypes might have been obscured by the lack of standardization in pre-analytical and analytical procedures across studies, and these differences were also a potential source of heterogeneity; finally, due to lack of appropriate data, the association of NLR and other clinical parameters, such as mean platelet volume, red cell distribution width, circulating tumor cells and gamma-glutamyl transferase was not explored. Thus, more worldwide studies are required to confirm the value of the NLR test for breast cancer diagnosis in the future.

In conclusion, elevated NLR is strongly associated with poor survival of breast cancer patients, and it can be regarded as a predictive and prognostic factor for patients with breast cancer. Further well designed prospective studies with multi-central and a large sample size are warrant to verify our findings.

## Conflict of interest statement

The authors declare no conflict of interest in this work.

## Author contributions

JC and QWD conceived and designed the experiments; YQP and BSH performed the experiments; HQY analyzed the data and helped in manuscript writing; HLS and XL contributed reagents and analysis tools; SKW and JC wrote the paper.

## Figures and Tables

**Table 1:** Main characteristics of eligible studies.

No. of studies	First author	Journal	Year	Country	Ethnicity	Specimens	Metastasis	Cut-off	Follow-up (month)	Number of patients	Analysis	Survival	HR estimation
[16]	Forget P	Ann Surg Oncol	2013	Belgium	Caucasian	Blood	Mix	3.4	NA	162,172	Univariate	RFS	HR + 95% CI
[22]	Azab B	Ann Surg Oncol	2012	USA	Caucasian	Blood	Mix	3.3	45.6(mean)	316	Multivariate	OS	HR + 95% CI
[23]	Noh H	J Breast Cancer	2013	Korea	Asian	Blood	No	2.5	6.1(mean)	442	Multivariate	CSS	HR + 95% CI
[24]	Forget P	Br J Anaesth	2014	Belgium	Caucasian	Blood	Mix	3.3	69.8(median)	720	Multivariate	OS,DFS	HR + 95% CI
[25]	Dirican A	Int J Clin Oncol	2014	Turkey	Asian	Blood	No	4	30(median)	1,527	Multivariate	OS,DFS	HR + 95% CI
[27]	Cihan YB	Asian Pac J Cancer Prev	2014	Turkey	Asian	Blood	No	3	Mean range 10 days-112 months	350	Univariate	OS,DFS	HR + 95% CI
[32]	Azab B	Med Oncol	2013	USA	Caucasian	Blood	Mix	3.3	60(mean)	437	Univariate	OS	HR + 95% CI
[33]	Nakano K	Anticancer Res	2014	Japan	Asian	Blood	Mix	2.5	85.8(mean)	167	Multivariate	DFS	HR + 95% CI

NA: not available; DFS: disease-free survival; OS: overall survival; RFS: recurrence-free survival; CSS: cancer-specific survival; HR: hazard ratio; CI: 95% confidence interval.

**Table 2:** Meta-analysis results.

Survival	Variables	Number of studies	Number of patients	*P* value			Regression model	
				*P*_H_	*P*_Z_	*P*_E_	Random	Fixed
OS	All	5	3,350	<0.001	0.031	0.894	2.28(1.08–4.80)	2.36(1.85–3.02)
	Ethnicity			<0.001				
	Asian	2	1,877	0.007	0.723	—	1.19(0.45–3.19)	1.46(1.06–2.02)
	Caucasian	3	1,473	0.096	<0.001	—	3.89(2.05–7.39)	4.53(3.11–6.60)
	Analysis method			0.263				
	Univariate	2	787	<0.001	0.495	—	2.11(0.25–17.88)	2.79(1.91–4.07)
	Multivariate	3	2,563	0.591	<0.001	—	2.10(1.52–2.89)	2.10(1.52–2.89)
	Metastasis			<0.001				
	No	2	1,877	0.007	0.723	—	1.19(0.45–3.19)	1.46(1.06–2.02)
	Mix	3	1,473	0.096	<0.001	—	3.89(2.05–7.39)	4.53(3.11–6.60)

DFS	All	4	2,764	0.050	0.093	0.762	1.41(0.94–2.12)	1.38(1.09–1.74)
	Ethnicity			0.141				
	Asian	3	2,044	0.06	0.328	—	1.27(0.78–2.06)	1.27(0.98–1.64)
	Caucasian	1	720	—	0.012	—	1.99(1.16–3.41)	1.99(1.16–3.41)
	Analysis method			0.010				
	Univariate	1	350	—	0.360	—	0.80(0.50–1.29)	0.80(0.50–1.29)
	Multivariate	3	2,414	0.545	<0.001	—	1.64(1.25–2.14)	1.64(1.25–2.14)
	Metastasis			0.053				
	No	2	1,877	0.044	0.732	—	1.11(0.62–1.99)	1.20(0.91–1.57)
	Mix	2	887	0.992	0.002	—	1.99(1.28–3.09)	1.99(1.28–3.09)

OS: overall survival; DFS: disease-free survival; *P*_H_, *P* value of heterogeneity test; *P*_Z_, *P* value of Z test; *P*_E_, *P* value of Egger’s test.

## References

[1] Siegel R., Naishadham D., Jemal A. (2013). Cancer statistics, 2013. CA Cancer J. Clin..

[2] Anders C.K., Johnson R., Litton J., Phillips M., Bleyer A. (2009). Breast cancer before age 40 years. Semin. Oncol..

[3] Assi H.A., Khoury K.E., Dbouk H., Khalil L.E., Mouhieddine T.H. (2013). Epidemiology and prognosis of breast cancer in young women. J. Thorac. Dis..

[4] DeSantis C., Ma J., Bryan L., Jemal A. (2014). Breast cancer statistics, 2013. CA Cancer J. Clin..

[5] Gabriel C.A., Domchek S.M. (2010). Breast cancer in young women. Breast Cancer Res..

[6] Ferguson N.L., Bell J., Heidel R., Lee S., Vanmeter S. (2013). Prognostic value of breast cancer subtypes, Ki-67 proliferation index, age, and pathologic tumor characteristics on breast cancer survival in Caucasian women. Breast J..

[7] Paul D., Kumar A., Gajbhiye A., Santra M.K., Srikanth R. (2013). Mass spectrometry-based proteomics in molecular diagnostics: discovery of cancer biomarkers using tissue culture. Biomed. Res. Int..

[8] Colotta F., Allavena P., Sica A., Garlanda C., Mantovani A. (2009). Cancer-related inflammation, the seventh hallmark of cancer: links to genetic instability. Carcinogenesis.

[9] Mantovani A., Allavena P., Sica A., Balkwill F. (2008). Cancer-related inflammation. Nature.

[10] Mantovani A., Romero P., Palucka A.K., Marincola F.M. (2008). Tumour immunity: effector response to tumour and role of the microenvironment. Lancet.

[11] Balkwill F., Mantovani A. (2010). Cancer and inflammation: implications for pharmacology and therapeutics. Clin. Pharmacol. Ther..

[12] Guthrie G.J., Charles K.A., Roxburgh C.S., Horgan P.G., McMillan D.C. (2013). The systemic inflammation-based neutrophil-lymphocyte ratio: experience in patients with cancer. Crit. Rev. Oncol. Hematol..

[13] Yao M., Liu Y., Jin H., Liu X., Lv K. (2014). Prognostic value of preoperative inflammatory markers in Chinese patients with breast cancer. Onco. Targets Ther..

[14] Satelli A., Brownlee Z., Mitra A., Meng Q.H., Li S. (2015). Circulating tumor cell enumeration with a combination of epithelial cell adhesion molecule- and cell-surface vimentin-based methods for monitoring breast cancer therapeutic response. Clin. Chem..

[15] Fentiman I.S., Allen D.S. (2010). Gamma-glutamyl transferase and breast cancer risk. Br. J. Cancer.

[16] Forget P., Machiels J.P., Coulie P.G., Berliere M., Poncelet A.J. (2013). Neutrophil:lymphocyte ratio and intraoperative use of ketorolac or diclofenac are prognostic factors in different cohorts of patients undergoing breast, lung, and kidney cancer surgery. Ann. Surg. Oncol..

[17] Hung H.Y., Chen J.S., Yeh C.Y., Changchien C.R., Tang R. (2011). Effect of preoperative neutrophil-lymphocyte ratio on the surgical outcomes of stage II colon cancer patients who do not receive adjuvant chemotherapy. Int. J. Colorectal Dis..

[18] Keizman D., Gottfried M., Ish-Shalom M., Maimon N., Peer A. (2012). Pretreatment neutrophil-to-lymphocyte ratio in metastatic castration-resistant prostate cancer patients treated with ketoconazole: association with outcome and predictive nomogram. Oncologist.

[19] Mallappa S., Sinha A., Gupta S., Chadwick S.J. (2013). Preoperative neutrophil to lymphocyte ratio >5 is a prognostic factor for recurrent colorectal cancer. Colorectal Dis..

[20] Sharaiha R.Z., Halazun K.J., Mirza F., Port J.L., Lee P.C. (2011). Elevated preoperative neutrophil:lymphocyte ratio as a predictor of postoperative disease recurrence in esophageal cancer. Ann. Surg. Oncol..

[21] Tomita M., Shimizu T., Ayabe T., Nakamura K., Onitsuka T. (2012). Elevated preoperative inflammatory markers based on neutrophil-to-lymphocyte ratio and C-reactive protein predict poor survival in resected non-small cell lung cancer. Anticancer Res..

[22] Azab B., Bhatt V.R., Phookan J., Murukutla S., Kohn N. (2012). Usefulness of the neutrophil-to-lymphocyte ratio in predicting short- and long-term mortality in breast cancer patients. Ann. Surg. Oncol..

[23] Noh H., Eomm M., Han A. (2013). Usefulness of pretreatment neutrophil to lymphocyte ratio in predicting disease-specific survival in breast cancer patients. J. Breast Cancer.

[24] Forget P., Bentin C., Machiels J.P., Berliere M., Coulie P.G. (2014). Intraoperative use of ketorolac or diclofenac is associated with improved disease-free survival and overall survival in conservative breast cancer surgery. Br. J. Anaesth..

[25] Dirican A., Kucukzeybek B.B., Alacacioglu A., Kucukzeybek Y., Erten C. (2014). Do the derived neutrophil to lymphocyte ratio and the neutrophil to lymphocyte ratio predict prognosis in breast cancer?. Int. J. Clin. Oncol..

[26] Neofytou K., Smyth E.C., Giakoustidis A., Khan A.Z., Cunningham D. (2014). Elevated platelet to lymphocyte ratio predicts poor prognosis after hepatectomy for liver-only colorectal metastases, and it is superior to neutrophil to lymphocyte ratio as an adverse prognostic factor. Med. Oncol..

[27] Cihan Y.B., Arslan A., Cetindag M.F., Mutlu H. (2014). Lack of prognostic value of blood parameters in patients receiving adjuvant radiotherapy for breast cancer. Asian Pac. J. Cancer Prev..

[28] Knobloch K., Yoon U., Vogt P.M. (2011). Preferred reporting items for systematic reviews and meta-analyses (PRISMA) statement and publication bias. J. Craniomaxillofac. Surg..

[29] Moher D., Liberati A., Tetzlaff J., Altman D.G., P. Group (2009). Preferred reporting items for systematic reviews and meta-analyses: the PRISMA statement. Ann. Intern. Med..

[30] DerSimonian R., Laird N. (1986). Meta-analysis in clinical trials. Control Clin. Trials.

[31] Mantel N., Haenszel W. (1959). Statistical aspects of the analysis of data from retrospective studies of disease. J. Natl. Cancer Inst..

[32] Azab B., Shah N., Radbel J., Tan P., Bhatt V. (2013). Pretreatment neutrophil/lymphocyte ratio is superior to platelet/lymphocyte ratio as a predictor of long-term mortality in breast cancer patients. Med. Oncol..

[33] Nakano K., Hosoda M., Yamamoto M., Yamashita H. (2014). Prognostic significance of pre-treatment neutrophil: lymphocyte ratio in Japanese patients with breast cancer. Anticancer Res..

[34] Hanahan D., Weinberg R.A. (2011). Hallmarks of cancer: the next generation. Cell.

[35] Templeton A.J., McNamara M.G., Seruga B., Vera-Badillo F.E., Aneja P. (2014). Prognostic role of neutrophil-to-lymphocyte ratio in solid tumors: a systematic review and meta-analysis. J. Natl. Cancer Inst..

[36] Corthay A. (2014). Does the immune system naturally protect against cancer?. Front. Immunol..

[37] Fridman W.H., Pages F., Sautes-Fridman C., Galon J. (2012). The immune contexture in human tumours: impact on clinical outcome. Nat. Rev. Cancer.

[38] Kawai O., Ishii G., Kubota K., Murata Y., Naito Y. (2008). Predominant infiltration of macrophages and CD8(+) T Cells in cancer nests is a significant predictor of survival in stage IV nonsmall cell lung cancer. Cancer.

[39] Rusakiewicz S., Semeraro M., Sarabi M., Desbois M., Locher C. (2013). Immune infiltrates are prognostic factors in localized gastrointestinal stromal tumors. Cancer Res..

[40] Lee P.P., Yee C., Savage P.A., Fong L., Brockstedt D. (1999). Characterization of circulating T cells specific for tumor-associated antigens in melanoma patients. Nat. Med..

[41] Kono H., Fujii H., Ogiku M., Hosomura N., Amemiya H. (2011). Role of IL-17A in neutrophil recruitment and hepatic injury after warm ischemia-reperfusion mice. J. Immunol..

[42] Kuang D.M., Zhao Q., Wu Y., Peng C., Wang J. (2011). Peritumoral neutrophils link inflammatory response to disease progression by fostering angiogenesis in hepatocellular carcinoma. J. Hepatol..

